# Comparative Genomic and Transcriptomic Analysis Reveals Specific Features of Gene Regulation in *Kluyveromyces marxianus*

**DOI:** 10.3389/fmicb.2021.598060

**Published:** 2021-02-26

**Authors:** Yao Yu, Wenjuan Mo, Haiyan Ren, Xianmei Yang, Wanlin Lu, Tongyu Luo, Junyuan Zeng, Jungang Zhou, Ji Qi, Hong Lu

**Affiliations:** ^1^State Key Laboratory of Genetic Engineering, School of Life Sciences, Fudan University, Shanghai, China; ^2^Shanghai Engineering Research Center of Industrial Microorganisms, Fudan University, Shanghai, China; ^3^National Technology Innovation Center of Synthetic Biology, Tianjin, China

**Keywords:** *Kluyveromyces marxianus*, *Saccharomyces cerevisiae*, fast growth, comparative analysis, ATP production, respiration chain, glucose transport

## Abstract

*Kluyveromyces marxianus* is a promising host for producing bioethanol and heterologous proteins. It displays many superior traits to a conventional industrial yeast species, *Saccharomyces cerevisiae*, including fast growth, thermotolerance and the capacity to assimilate a wider variety of sugars. However, little is known about the mechanisms underlying the fast-growing feature of *K. marxianus*. In this study, we performed a comparative genomic analysis between *K. marxianus* and other Saccharomycetaceae species. Genes involved in flocculation, iron transport, and biotin biosynthesis have particularly high copies in *K. marxianus*. In addition, 60 *K. marxianus* specific genes were identified, 45% of which were upregulated during cultivation in rich medium and these genes may participate in glucose transport and mitochondrion related functions. Furthermore, the transcriptomic analysis revealed that under aerobic condition, normalized levels of genes participating in TCA cycles, respiration chain and ATP biosynthesis in the lag phase were higher in *K. marxianus* than those in *S. cerevisiae*. Levels of highly copied genes, genes involved in the respiratory chain and mitochondrion assembly, were upregulated in *K. marxianus*, but not in *S. cerevisiae*, in later time points during cultivation compared with those in the lag phase. Notably, during the fast-growing phase, genes involved in the respiratory chain, ATP synthesis and glucose transport were co-upregulated in *K. marxianus*. A few shared motifs in upstream sequences of relevant genes might result in the co-upregulation. Specific features in the co-regulations of gene expressions might contribute to the fast-growing phenotype of *K. marxianus*. Our study underscores the importance of genome-wide rewiring of the transcriptional network during evolution.

## Introduction

For decades, microorganisms have been used as cell factories to produce industrial compounds, including biofuels, bulk chemicals, pharmaceuticals, and heterologous proteins. *Saccharomyces cerevisiae* is the most commonly used eukaryotic cell factory, thanks to its safe status, ease of genetic manipulation and a wealth of genomic, genetic, and biochemical knowledge (Nielsen and Jewett, 2008). However, other non-conventional yeasts possess desirable features for niche applications and can provide several advantages over *S. cerevisiae*. Such yeasts have the potential to become the next-generation cell factories. *Kluyveromyces marxianus* is one such alternative yeast.

*Kluyveromyces marxianus* is a homothallic, hemiascomycete yeast commonly isolated in dairy products and fruits. It belongs to the Saccharomycetaceae family and is phylogenetically related to *S. cerevisiae* with many superior traits. For examples, *K. marxianus* can assimilate inulin, lactose and pentose sugars such as xylose and arabinose, which can not be utilized by *S. cerevisiae*. *K. marxianus* is more thermotolerant, as it can grow at a temperature up to 52 degrees (Lane and Morrissey, 2010; Gombert et al., 2016). Notably, *K. marxianus* is the fastest-growing eukaryote known so far (Groeneveld et al., 2009). The fastest growth rate of *K. marxianus* is 0.80 h^–1^, that of *K. lactis* is 0.50 h^–1^ (Lane and Morrissey, 2010), that of *S. cerevisiae* is 0.37 h^–1^ (Lane and Morrissey, 2010), and that of *Pichia pastoris* is 0.18 h^–1^ (Gao et al., 2012). Given its GRAS status and desirable traits, *K. marxianus* emerges as a promising host for producing bioethanol and heterologous proteins (Zoppellari and Bardi, 2013; Gombert et al., 2016; Mo et al., 2019). However, less is known about mechanisms underlying the desirable traits of *K. marxianus*, especially mechanisms of the fast-growing feature.

*Kluyveromyces marxianus* strains show a high level of genetic and phenotypic variation (Ortiz-Merino et al., 2018). Two *K. marxianus* strains have been completely sequenced and annotated, including DMKU 3-1042 (Lertwattanasakul et al., 2015), and NBRC1777 (Inokuma et al., 2015), while genomes of KCTC 17555 (Jeong et al., 2012), DMB1 (Suzuki et al., 2014), and CCT 7735 (Silveira et al., 2014) were draft sequences. Transcriptomic studies of *K. marxianus* were mainly focused on its physiological properties, such as xylose catabolism (Schabort du et al., 2016), high-temperature resistance (Lertwattanasakul et al., 2015), and ethanol tolerance (Mo et al., 2019). Few comparative transcriptomic studies have been performed between *K. marxianus* and other species in the Saccharomycetaceae family. For example, analysis of overall metabolic turnover and transcript dynamics in glycolysis and the TCA cycle revealed the difference in adaptive pyruvate metabolic response between *K. marxianus* and *S. cerevisiae* during aerobic growth (Sakihama et al., 2019).

FIM1 is a *K. marxianus* strain developed by for industrial applications. High yield of ethanol, vaccines and lignocellulolytic enzymes was achieved in the FIM1 strain (Zhou et al., 2018; Duan et al., 2019; Mo et al., 2019). In this study, we carried out a complete genome sequencing and annotation of the *K. marxianus* FIM1 strain. A comparative genomic analysis between *K. marxianus* and Saccharomycetaceae species was performed to characterize species-specific genes and particularly highly copied genes in *K. marxianus*. A comparative transcriptomic analysis was also performed between the *K. marxianus* and *S. cerevisiae* cells grown in the rich medium under aerobic condition. Results suggested that compared to *S. cerevisiae*, *K. marxianus* displayed increased expressions of genes participating in TCA cycles, respiration chain and ATP biosynthesis in the lag phase. During the exponential phase, *K. marxianus* exhibited a tighter co-upregulation of genes involved in the respiratory chain with those involved in ATP synthesis and glucose transport. The specific features of gene regulation in *K. marxianus* might shed light on the mechanisms underlying the fast growth feature of this species.

## Materials and Methods

### Yeast Strains and Culture

*Kluyveromyces marxianus* FIM1 strain used in this work was deposited at China General Microbiological Culture Collection Center (CGMCC, No 10621). *S. cerevisiae* S288c strain was used. FIM1 and S288c were growing in YPD liquid medium (2% glucose, 2% peptone, 1% yeast extract) under aerobic condition at 30°C.

### Genome Sequencing, Assembly and Annotation

Nuclear DNA was extracted from an overnight culture of *K. marxianus* FIM1 by a Yeast Genomic DNA Extraction Kit (D1900, Solarbio, China). Contigs were assembled using 300-bp pair-end reads by SOAPdenovo (Luo et al., 2012), then 3-kb and 8-kb mate-pair reads were used to construct scaffolds using program space, which resulted in eight scaffolds. The gaps in each scaffold were filled using long reads produced by PacBio RSII, and the low-quality bases were corrected by mapping 300-bp pair-end reads on the scaffolds. Protein-coding genes were predicted from the assembled genome by using the “Yeast Genome Annotation Pipeline” (Proux-Wera et al., 2012), and then blasted against NCBI non-redundant (nr) protein database with *E*-value cutoff as 10^–5^ for functional annotation. tRNAs were predicted by tRNAscan-SE (Lowe and Eddy, 1997). rRNAs were predicted by Barrnap^1^. Genome sequence and annotation GFF3 files of FIM1 were deposited in the NCBI with GenBank accession No. CP015054–CP015061 and assembly accession GCA_001854445.2.

Annotations of DMB1 (GenBank assembly accession GCA_000747785.1), KCTC 17555 (GenBank assembly accession GCA_000299195.2), and CCT 7735 (GenBank accession CP009303–CP009311) were performed as described above. Annotation GFF files of DMB1, KCTC 17555, and CCT 7735 genome were provided as Supplementary Files 1–3.

### Identification of Gene Families and the Phylogenetic Analysis

Genome of *Kluyveromyces lactis* NRRL Y-1140 (GenBank assembly accession GCA_000002515.1), *Kluyveromyces aestuarii* ATCC18862 (GCA_000179355.1), *Eremothecium cymbalariae* DBVPG7215 (GCA_000235365.1), *Eremothecium gossypii* ATCC10895 (GCA_000091025.4), *Lachancea thermotolerans* CBS6340 (GCA_000142805.1), *Naumovozyma castellii* CBS4309 (GCA_000237345.1), *Naumovozyma dairenensis* CBS421 (GCA_000227115.2), *Kazachstania africana* CBS2517 (GCA_000304475.1), *Saccharomyces cerevisiae* S288c (GCA_000146045.2), *Tetrapisispora phaffii* CBS4417 (GCA_000236905.1), *Torulaspora delbrueckii* CBS1146 (GCA_000243375.1), *Zygosaccharomyces rouxii* CBS732 (GCA_000026365.1), *Pichia pastoris* GS115 (GCA_001708105.1), and *Yarrowia lipolytica* CLIB122 (GCA_000002525.1) were downloaded from NCBI and protein sequences were extracted. First, an all-against-all BLAST was applied to these protein sequences with *E*-values < 10^–5^. Second, global protein similarities were calculated using InParanoid (O’Brien et al., 2005), and those matched with both sufficient gene coverage (>75%) and alignment identity (>50%) were left for further analyses. Third, ortholog groups, i.e., the ‘gene families,’ were identified by comparing protein alignments using OrthoMCL (Li et al., 2003). Multiple sequence alignment of proteins in each gene family was obtained using MUSCLE with default parameters (Edgar, 2004), and was further trimmed with trimAL (Capella-Gutierrez et al., 2009). Gene families containing a single copy of genes within each genome were concatenated into a supergene for the reconstruction of a phylogenetic tree, using the maximum likelihood method with 100 bootstrap replicates.

### Analysis of Gene Copy Number

In a gene family of *K marxianus*, genes with the same functional annotation were considered as replicated genes and the copy number was counted. Functional annotation was performed by using the “Yeast Genome Annotation Pipeline” as described above. After families containing replicated genes were identified in *K. marxianus*, copy numbers of replicated genes in the corresponding families of other Saccharomycetales species were determined.

### Preparation of Samples for RNA-Seq

*Kluyveromyces marxianus* and *Saccharomyces cerevisiae* cells were grown in 50 mL YPD liquid medium overnight. Cells were transferred into 50 mL fresh YPD liquid medium to start an OD_600_ of 0.1. After that, cells from 1 to 5 mL cultures were collected at 1, 4, 6, 12, 24, 48, and 72 h after culturing in rich medium. Samples were collected from separate cultures for biologic repeats. Total RNA was extracted using the ZR Fungal/Bacterial RNA MiniPrepTM (Zymo Research, CA). RNAseq was performed in Genergy Biotechnology company (Shanghai, China).

### RNA-Seq Analysis

15.1 million pair-end reads on average for each RNA sample were obtained. After initial QC, short 150 bp reads were mapped to the reference genomes of *K. marxianus* FIM1 and *S. cerevisiae* S288c (Saccharomyces Genome Database) using HISAT2.1 (Kim et al., 2015). The expression level of a family was the sum of FPKM values of genes in the family. The level of a gene family at a different time point after the culturing was compared with that at 1 h and the change was calculated by using the GFOLD algorithm. GFOLD algorithm is roughly equivalent to the raw fold change log_2_ratio value but takes into account the uncertainty of gene expression measurement by RNA-seq, thus is more reliable than the raw fold change (Feng et al., 2012). Two-fold or more changes, i.e., |GFOLD| ≥1, were defined as significantly differentially expressed. A change with GFOLD > = 1 was defined as up-regulation and a change with GFOLD ≤ –1 was referred to as downregulation.

### RT-qPCR

To analyze mRNA levels in lag phase, an overnight culture of *K. marxianus* or *S. cerevisiae* was transferred into 50 mL fresh YPD liquid medium to start an OD_600_ of 0.1 and cells were collected after 1 h. To analyze changes in mRNA levels upon heat stress, an overnight culture of *K. marxianus* was transferred into 50 mL fresh YPD liquid medium to start an OD_600_ of 0.1 and cells were collected after growing at 45 degrees or 30 degrees for 8 h. Total RNAs were isolated from three independent cultures and reverse-transcribed into cDNA by PrimeScript RT (RR037A, Takara). qPCR was performed on a LightCycler 480 (Roche Applied Science, Germany) with the SYBR Premix Ex Taq II (RR820A, Takara). The mRNA level of the individual gene was normalized against the average level of *MPE1*, *TRK1*, and *SWC4*. Primers used for RT-qPCR were listed in Supplementary Table 1.

### Prediction of the Function of *K. marxianus*-Specific (KMS) Families Based on the Correlation Coefficient

Inspired by the idea of using gene co-expression network to predict the function of hypothetical genes in *Aspergillus nige*r (Schape et al., 2019), Pearson correlation coefficient (denoted as *r*) was applied to infer the function of KMS families. For each gene family, its expression level was the sum of FPKM values of genes contained in the family. Value of *r* was calculated between a KMS family and a gene with known function at different time points after culturing. If *r* > 0.8, the function of the known gene can be considered as the candidate function of the KMS family. The known gene with the highest *r* value was chosen for the predicted function.

## Results

### Comparative Genomic Analysis Reveals Species-Specific and Highly Copied Genes of *K. marxianus*

A genome sequencing of the *K. marxianus* FIM1 strain was performed in this study. Eight nuclear chromosome sequences were obtained with a total size of 10.9 Mbp. The genome was annotated via “Yeast Genome Annotation Pipeline” for gene identification and via non-redundant (nr) database for functional annotation (Proux-Wera et al., 2012). Five thousand two hundred and two genes were systematically identified in the FIM1 strain, higher than previously reported 4952 genes identified in the DMKU 3-1042 strain (Lertwattanasakul et al., 2015). Two copies of rRNA were located in chromosome 5. The correlations between the systematic gene names in both strains were listed in Supplementary Table 2. Coding sequences of genes in FIM1 were listed in Supplementary Table 3. GFF3 files were uploaded in the NCBI Genome database (accession numbers CP015054–CP015061 and assembly accession GCA_001854445.2).

**FIGURE 1:**
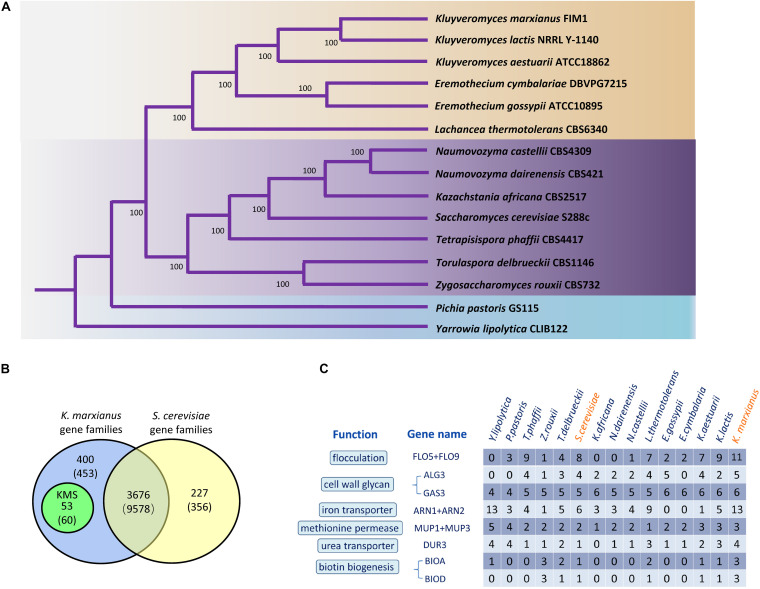
Gene families and highly copied genes of *K. marxianus.* **(A)** Phylogenetic tree of *K. marxianus* evolution in Saccharomycetales. The tree was generated based on the concatenated sequence of 636 single-copy genes without long-branch score heterogeneity. The bootstrap value 100 at each node point indicates the correctness of inferred topology. The blue part contains *Y. lipolytica* in Dipodascaceae and *P. pastoris* in Phaffomycetaceae as outgroups. The purple and the brown parts represent two major clades in Saccharomycetaceae, respectively, i.e., the one close to *Saccharomyces* and the one close to *Kluyveromyces*. **(B)** Gene families in *K. marxianus* FIM1 and *S. cerevisiae* S288C. Genomes of 15 yeast species in Figure 1A was used to identify gene families as described in methods. The number of genes included in the families is given in parentheses. Genes included in the families in *K. marxianus* and *S. cerevisiae* were shown in Supplementary Table 4. **(C)** Comparison of gene copy numbers among 15 Saccharomycetales species.

To identify *K. marxianus*-specific genes, a concept of “gene family” was introduced. Each gene family is a set of homologous genes among 15 yeast Saccharomycetales species, including 13 species in Saccharomycetaceae, one in Phaffomycetaceae and one in Dipodascaceae as outgroups. Totally, 636 gene families contain a single copy of a gene in each species. Single-copy genes were concatenated in each species into a supergene, followed by reconstructing a phylogenetic tree using the maximum likelihood method (Figure 1A). Our results are consistent with a previous report (Wolfe et al., 2015).

There are 4076 families in *K. marxianus* and 3903 families in *S. cerevisiae*, in which 3676 gene families contain genes from both species (Figure 1B and Supplementary Table 4). Notably, 53 gene families only contain *K. marxianus* genes and no gene from other species in Saccharomycetale. These 53 families contained 60 genes in total and these genes were named as KMS (*K. marxianus*-specific) genes (Figure 1B and Supplementary Table 5). Four KMS families contain more than one gene, and the rest 49 KMS families contained a single gene. Five KMS genes were located inside 10 kb from the telomere.

The existence of KMS genes in other *K. marxianus* strains, including DMKU3-1042, NBRC1777, DMB1, CCT 7735 and KCTC17555 was investigated (Supplementary Table 5). Briefly, 36 and 47 KMS genes were systematically annotated in DMKU3-1042 and NBRC1777 strains respectively. Sequences of the rest KMS genes could be found in the genome of these strains, but they were not annotated. Genomes of DMB1, CCT 7735, and KCTC 17555 were draft sequences. Annotations of these genomes were performed in this study as described in methods. Briefly, 56, 58, and 58 KMS genes were annotated in KCTC17555, DMB1 and CCT 7735 strains respectively, while the rest of KMS genes could be found without annotation. FIM1_4308 was missing in CCT 7735 genome and FIM1_2658 was missing in DMB1, presumably due to sequencing error. In sum, a complete set of KMS genes exists in different *K. marxianus* strains, except two cases mentioned above. Therefore, KMS genes are not FIM1-specific genes but are truly species-specific genes of *K. marxianus*.

The copy number of genes in different yeast species were compared (Figure 1C). Genes involved in flocculation, iron transport and biotin biosynthesis have particularly high copies in *K. marxianus*, i.e., 11, 13, and 3, respectively (Supplementary Table 6). One copy of FLO5/FLO9 gene, two copies of ARN1/ARN2 gene, two copies of BIOA and two copies of BIOD were located inside 10 kb from a telomere in FIM1. Highly copied genes may contribute to specific features of *K. marxianus*.

### Functions of KMS Families

**FIGURE 2:**
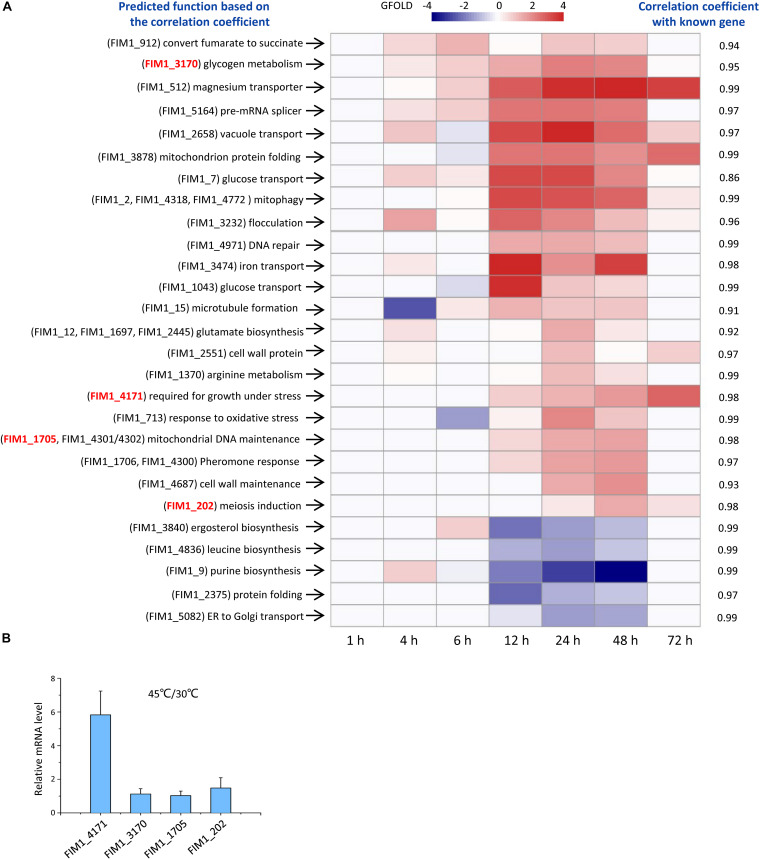
Functions of KMS genes. **(A)** Expressions and predicted functions of KMS genes. *K. marxianus* cells were collected at 1, 4, 6, 12, 24, 48, and 72 h after culturing in the YPD medium. Expression levels of KMS families at later time points were compared with those at 1 h. KMS families that exhibited twofold or more changes were shown here. A color bar corresponding to GFOLD values was shown on the top. Predicted functions of KMS families were listed on the left and corresponding correlation coefficients were on the right. Genes inside the family were shown in the parentheses. Genes selected for investigation upon heat stress in **(B)** were highlighted in red. **(B)** Changes in the mRNA levels of selected KMS genes upon heat stress. mRNA level of a KMS gene in cells grown at 45 degrees was compared with the average mRNA level in cells grown at 30 degrees. The value represents the mean ± SD from three independent repeats.

Out of the 53 KMS families, 49 families have not been functionally annotated. The function of each KMS family was predicted based on the similarity between its expression and the expressions of genes with known functions. *K. marxianus* cells were collected at 1, 4, 6, 12, 24, 48, and 72 h after culturing in a rich YPD medium and subjected to RNA-seq. The expression level of a gene family is the sum of FPKM values of the gene(s) in the family. By using the expression level of a gene family at 1 h as control, changes of the expression level of the family at later time points were calculated using the GFOLD algorithm. Expression levels of 22 KMS families were upregulated and those of 5 KMS families were downregulated at later time points (Figure 2 and Supplementary Table 7). Upregulated KMS families contained 29 genes, which occupied approximately 48% of total KMS genes. Pearson correlation coefficient (denoted as *r*) was calculated between a KMS family and a gene with known function, based on the hypothesis that strongly co-expressed genes are likely to function in the same or closely related biological pathways (Dutkowski et al., 2013; Schape et al., 2019). The known gene with the highest *r* value (at least above 0.8) were used to infer the possible function of a KMS family. The predicted functions of KMS families and corresponding *r* values were listed in Figure 2A. Results suggested that upregulated KMS families mainly participated in glycogen metabolism, glucose transport and mitochondrial related function, while the downregulated KMS families might be involved in ergosterol, leucine, and purine biosynthesis.

To demonstrate the reliability of prediction based on co-expression, changes in mRNA levels of selected KMS genes upon heat stress were investigated. FIM1_4171 was predicted to be required for the growth under stress. The mRNA level of FIM1_4171 increased by fivefold after growing in 45 degrees (Figure 2B). On the other hand, the mRNA levels of FIM1_3170, FIM1_1705 and FIM1_202, which were predicted to play role in irrelevant pathways, were kept constant upon heat stress (Figure 2B).

### Clustering of Gene Families in *K. marxianus* and *S. cerevisiae* by Their Expression Patterns

**FIGURE 3:**
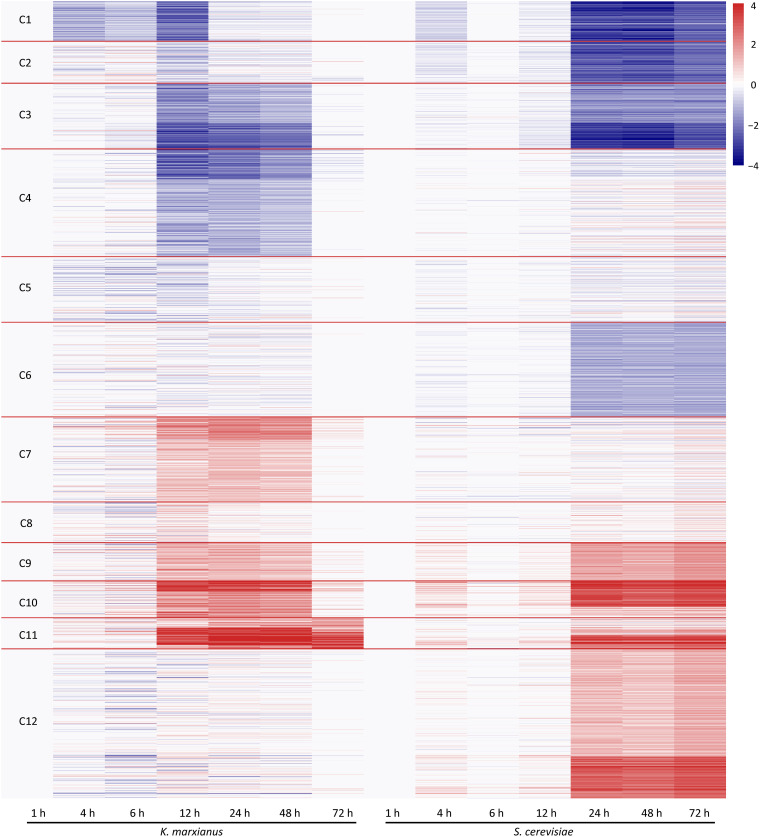
Clustering of differentially expressed families of *K. marxianus and S. cerevisiae* by their expression patterns. Each horizontal line represents a gene family shared by *K. marxianus* and *S. cerevisiae*. The color of a segment of a line is corresponding to the GFOLD value of a gene family at each time point respective to the value at 1 h. Using k-means based on Euclidean distance, gene families were grouped into 12 clusters (C1∼C12) by their expression patterns.

To perform a comparative transcriptomic analysis between *K. marxianus* and *S. cerevisiae*, *S. cerevisiae* cells were also collected at 1, 4, 6, 12, 24, 48, and 72 h after culturing in the YPD, and then subjected to the RNA-seq. Gene family contains a set of homologous genes and is suitable for many-to-many comparison between *K. marxianus* and *S. cerevisiae*. There were 3676 gene families that contained both *K. marxianus* and *S. cerevisiae* genes (Figure 1B). Out of them, 2535 gene families were identified as differentially expressed in at least one time points after 1 h in either *K. marxianus* or *S. cerevisiae*, by using the expression values of families at respective 1 h as control. These 2535 families were then grouped into 12 clusters according to their expression patterns (C1∼C12) (Figure 3), using the k-means method calculated on Euclidean distance. Relative expression levels of gene families in each cluster were listed in Supplementary Table 8. A summary of significantly enriched GO terms (*p*-value < 0.05) in each cluster was listed in Table 1.

**TABLE 1:** Summary of significantly enriched GO terms in 12 clusters in which differentially expressed families of *K. marxianus* and *S. cerevisiae* were grouped by their expression patterns.

Cluster	Number of gene families	Representative Enriched GO terms
1	128	Ribosome biogenesis, tRNA processing, valine biosynthesis, pre-replicative complex assembly involved in DNA replication
2	132	Ribosome biogenesis, tRNA processing, translation, glycolysis, cell wall formation, amino acid catabolism
3	209	Translation, protein folding, purine biosynthesis, fatty acid biosynthesis, steroid biosynthesis, cytokinesis
4	344	Protein glycosylation, DNA replication, nucleotide biosynthesis, cell cycle, lipid metabolism, vesicle transport
5	208	RNA processing, iron metabolism, ribosome biogenesis, protein glycosylation, kinetochore assembly, mitosis
6	302	DNA replication, amino acid degradation, glycogen biogenesis, glycolysis, respiratory chain, DNA repair, vacuole transport
7	271	Biotin biosynthesis, amino acid transport, respiratory chain, flocculation, iron transport, mitochondrion assembly
8	128	Glucose transport, ATP biogenesis, TCA cycle, endocytosis, autophagy, sporulation, proton transport
9	120	Flocculation, fructose metabolism, trehalose biosynthesis, autophagy, mitophagy, TCA cycle, respiratory chain, cell aging
10	116	Xylose catabolism, glycogen biosynthesis, TCA cycle, response to oxidative stress, autophagy, apoptosis
11	97	Acetate transport, ammonium transport, acetate biosynthesis, NADPH regeneration, fatty acid beta-oxidation
12	480	intracellular signal transduction, MAPK cascade, cell wall organization, osmosensory signaling

*Significantly enriched GO terms (*p*-value < 0.05) were selected. A full list of selected GO terms was shown in Supplementary Table 9.*

As illustrated in Figure 3, at the early stage of cultivation (4 and 6 h), there was no dramatic change of expression in the clusters. Expressions of *K. marxianus* and *S. cerevisiae* families showed dramatic changes since 12 and 24 h, respectively. The time points were in line with the late exponential phase, as showed by the growth curves of *K. marxianus* and *S. cerevisiae* (Supplementary Figure 1).

Several clusters displayed similar patterns of expressions in *K. marxianus* and *S. cerevisiae*. The expression levels of genes involved in ribosome biogenesis and protein translation, clustered in C1∼C3, were substantially downregulated in both species at later time points comparing to those at 1 h (Figure 3 and Table 1). Genes participating in trehalose biosynthesis, TCA cycle, respiratory chain and autophagy, clustered in C9∼C11, were upregulated after 1 h in both species (Figure 3 and Table 1). However, the expression patterns in the clusters of C4, C6, C7, and C12 were different between *K. marxianus* and *S. cerevisiae*. In C12, genes implicated in the intracellular signal transduction and MAPK cascade were kept unchanged in *K. marxianus*, while they were upregulated in *S. cerevisiae* (Figure 3 and Table 1). In C4, genes involved in DNA replication and cell cycle were downregulated in *K. marxianus* and were hardly changed in *S. cerevisiae* (Figure 3 and Table 1).

One major difference between *S. cerevisiae* and *K. marxianus* is their different Crabtree characters. *S. cerevisiae*, a Crabtree-positive species, produces ethanol under aerobic conditions when glucose is in excess (Pronk et al., 1996). As an inherent Crabtree-negative species, *K. marxianus* grows faster under aerobic conditions without ethanol fermentation (Fonseca et al., 2008). *K. marxianus* enhances metabolic turnover in the TCA cycle during aeration, which was proposed to promote efficient electron flux and respiration (Sakihama et al., 2019). Consistent with this idea, as shown in the C7 group in this study, genes involved in the respiratory chain and mitochondrion assembly, were upregulated in *K. marxianus* but kept unchanged in *S. cerevisiae* (Figure 3 and Table 1). In addition, genes involved in biotin biosynthesis, iron transport, flocculation were also specifically upregulated in *K. marxianus* (Figure 3 and Table 1). Interestingly, these genes were overlapped with highly copied genes in *K. marxianus* (Figure 1C), suggesting that increased expressions of highly copied genes during cultivation might contribute to specific traits of *K. marxianus*.

### Comparing to *S. cerevisiae*, *K. marxianus* Exhibited Higher Expressions of Genes Involved in the TCA Cycle, Respiratory Chain and ATP Synthesis in the Lag Phase

**FIGURE 4:**
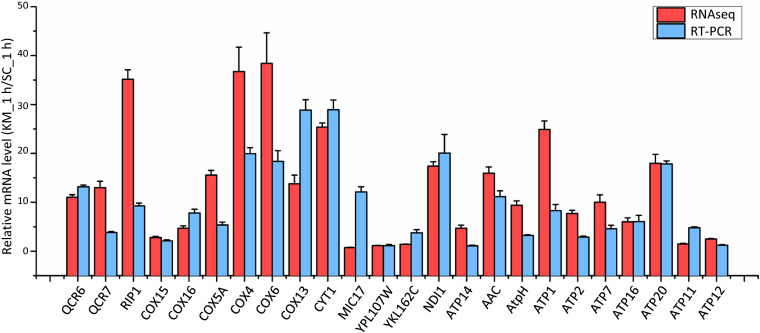
Comparison of mRNA levels of the respiratory chain and ATP synthesis genes in *K. marxianus* and *S. cerevisiae* after growing 1 h. The mRNA level of an indicated *K. marxianus* gene was normalized by the average mRNA level of three housekeeping genes, including *MPE1*, *TRK1*, and *SWC4*. Similarly, the mRNA level of an indicated *S. cerevisiae* gene was normalized. Normalized mRNA level of a *K. marxianus* gene was compared with the average of normalized mRNA levels of the *S. cerevisiae* gene from three repeats. Relative mRNA level (KM_1 h/SC_1 h) was shown by a red column (data obtained by RNAseq) or a blue column (data obtained by RT-PCR). The value represents the mean ± SD from three independent repeats.

*Saccharomyces cerevisiae* and *Kluyveromyces marxianus* cells were in the lag phase after being cultivated in the rich medium for 1 h. At this time point, 357 and 437 gene families in *K. marxianus* displayed higher and lower levels of expressions, respectively comparing to the equivalent families in *S. cerevisiae* (Supplementary Table 10). Representative GO terms enriched in differentially expressed families were listed in Table 2 and a full list of enriched GO terms was shown in Supplementary Table 11. Compared to *S. cerevisiae*, *K. marxianus* displayed enhanced expressions of genes implicated in TCA cycle, respiratory chain and ATP synthesis, meanwhile displayed reduced expressions of genes involved in the amino acid transport and cellular drug response. Enhanced expressions of *K. marxianus* genes involved in the respiratory chain and ATP synthesis during lag phase were validated by RT-qPCR analysis (Figure 4). The result was consistent with a strong tendency for respiration in Crabtree-negative *K. marxianus* (Gombert et al., 2016).

**TABLE 2:** Summary of significantly enriched GO terms in the families of *K. marxianus* whose expressions levels at 1 h were different from those of equivalent families of *S. cerevisiae.*

Type	Number of gene families	Representative enriched GO terms
Upregulated	357	TCA cycle, flocculation, aerobic respiration, ATP synthesis, mitochondrial transport, mitochondrial translation
Downregulated	437	Cellular response to drug, amino acid transport, lysine biosynthesis, ribosome biogenesis, fatty acid catabolism

*Significantly enriched GO terms (*p*-value < 0.05) were selected. A full list of selected GO terms was shown in Supplementary Table 11.*

### Comparison of Changes in Gene Expressions During the Fast-Growing Phase in *K. marxianus* and *S. cerevisiae*

**FIGURE 5:**
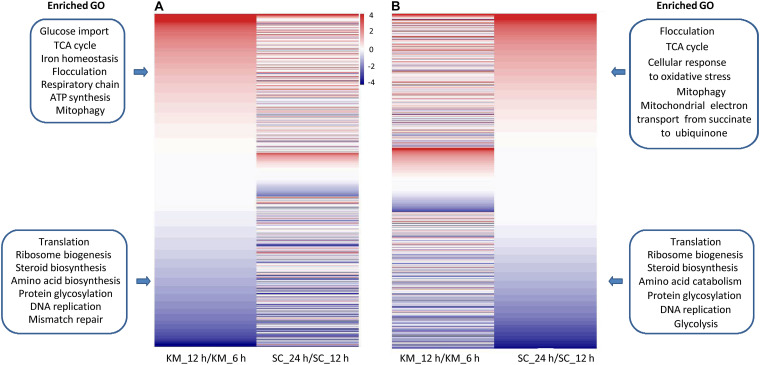
Comparison of changes in gene expression during the fast-growing phase in *K. marxianus* and *S. cerevisiae* **(A)** Families of *K. marxianus* ordered by GFOLD values. Corresponding families of *S. cerevisiae* were listed aside. GFOLD values of *K. marxianus* families indicate relative expression levels at 12 h after culturing comparing to those at 6 h (KM_12 h/KM_6 h). GFOLD values of *S. cerevisiae* families indicate relative expression levels at 24 h comparing to those at 6 h (SC_24 h/SC_12 h). Representative enriched GO terms in upregulated families and downregulated families of *K. marxianus* were shown in the upper box and lower box respectively. **(B)** Families of *S. cerevisiae* ordered by GFOLD values. Corresponding families of *K. marxianus* were listed aside. Representative enriched GO terms in *S. cerevisiae* families were shown in the boxes as **(A)**.

The fast-growing phase of *K. marxianus* was between 6 and 12 h, and that of *S. cerevisiae* was between 12 and 24 h (Supplementary Figure 1). The comparison of changes in gene expressions during this phase between two species was performed. In the case of *K. marxianus*, expression levels of the families at 12 h were compared with those at 6 h and changes in expression levels were calculated as GFOLD values. Similarly, expression levels of the families in *S. cerevisiae* at 24 h were compared with those at 12 h. When GFOLD values of *K. marxianus* families were ordered from high to low, GFOLD values of corresponding *S. cerevisiae* families also displayed a generally high-to-low pattern (Figure 5A and Supplementary Table 12). Vice versa, when the families of *S. cerevisiae* were ordered by their GFOLD values, GFOLD values of corresponding *K. marxianus* families followed a generally high-to-low pattern (Figure 5B and Supplementary Table 12). Results suggested that two species shared a generally similar tendency of changes in the expressions of homologous families during the fast-growing phase. Genes from the upregulated families and downregulated families were subjected to a GO analysis individually (Supplementary Table 13). Representative enriched GO terms of *K. marxianus* and *S. cerevisiae* families were shown in the boxes of Figures 5A,B, respectively. The comparison of enriched GO terms between two species was listed in Table 3. Genes involved in the TCA cycle and respiratory chain were both upregulated in *K. marxianus* and *S. cerevisiae*, while cell cycle-related genes exhibited little expression change in either species. Genes involved in glucose transport, iron homeostasis and ATP biosynthesis were upregulated in *K. marxianus*, while they were kept unchanged in *S. cerevisiae*. Genes involved in protein folding and response to heat were kept unchanged in *K. marxianus*, while they were upregulated in *S. cerevisiae*.

**TABLE 3:** Comparison of changes in gene expressions during the fast-growing phase in *K. marxianus* and *S. cerevisiae*.

Trend	Type	Number of families	Enriched GO terms
**Consistent**	**KM ↑ vs. SC ↑**	229	Flocculation, TCA cycle, autophagy, mitochondrial electron transport, xylose catabolism, response to ROS
	**KM ↓ vs. SC ↓**	330	Translation, ribosome biogenesis, tRNA processing, sterol biosynthesis, fatty acid biosynthesis
	**KM – vs. SC –**	1072	Phosphorylation, amino acid transport, cell cycle, biotin biosynthesis, DNA repair, osmosensory signaling
**Inconsistent**	**KM ↑ vs. SC –**	368	Glucose transport, iron ion homeostasis, ATP biosynthesis, respiratory chain complex IV assembly
	**KM ↑ vs. SC ↓**	24	Glycine catabolism, IMP biosynthesis, mitochondrial proton-transporting ATP synthase complex assembly
**Inconsistent**	**KM ↓ vs. SC –**	321	Protein glycosylation, steroid biosynthesis, DNA replication, ER to Golgi vesicle-mediated transport
	**KM ↓ vs. SC ↑**	44	Lactate catabolism, lipid storage, phospholipid biosynthesis, response to pheromone
**Inconsistent**	**KM – vs. SC ↑**	344	Ammonium transport, protein folding, pentose-phosphate shunt, response to heat
	**KM – vs. SC ↓**	329	Ribosome biogenesis, rRNA processing, translation, cell wall organization, NADH oxidation

*KM stands for *K. marxianus* families and SC for *S. cerevisiae* families.*

*‘↑,’ ‘↓,’ and ‘–’ denote up-regulation, down-regulation, and no change during the fast-growing phase, respectively.*

*Families included in each section were shown in Supplementary Table 14.*

### During the Fast-Growing Phase, Upregulation of Genes Involved in the Respiratory Chain Was Coupled With That of Genes Involved in ATP Synthesis and Glucose Transport in *K. marxianus*

**FIGURE 6:**
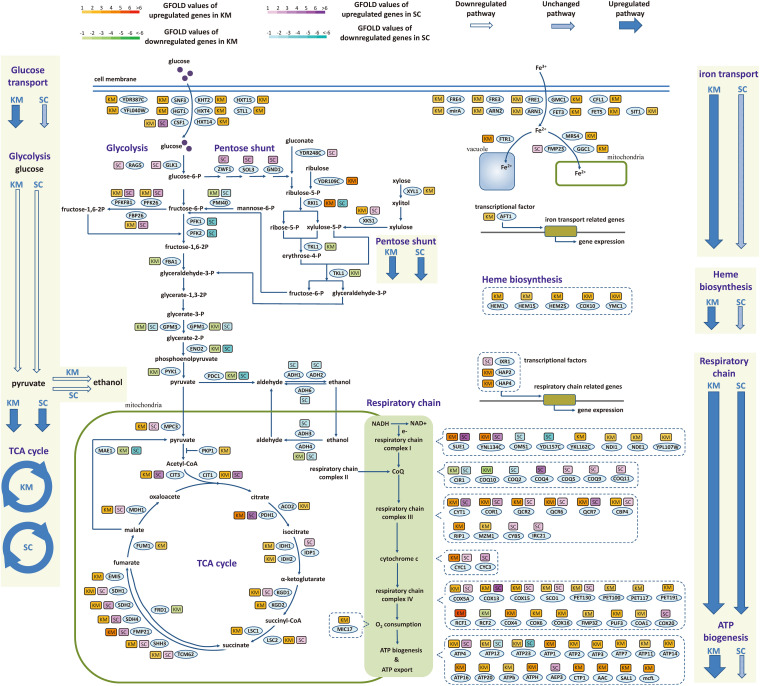
Expression changes of genes involved in central metabolic pathways during the fast-growing phase in *K. marxianus* and *S. cerevisiae*. The color of a box nearby a gene indicates the change of its expression during the fast-growing phase in *K. marxianus* (KM) and *S. cerevisiae* (SC). In the case of *K. marxianus*, expression levels of genes at 12 h after culturing were compared with those at 6 h and changes in expression levels were calculated as GFOLD values. Similarly, expression levels of *S. cerevisiae* genes at 24 h were compared with those at 12 h. Color bars corresponding to GFOLD values were shown on the top. Hollow arrow, arrow filled with slant pattern and blue solid arrow represent generally downregulated pathway, unchanged pathway and upregulated pathway, respectively.

The difference between two species in the regulation of central metabolic genes during the fast-growing phase was investigated in detail. As shown in Figure 6, the central metabolism was divided into material metabolism (left part, including glucose transport, glycolysis, and TCA cycle) and energy metabolism (right part, including respiratory chain, ATP synthesis, iron transport, and heme biosynthesis). In material metabolism, out of 10 genes involved in glucose transport, nine genes were upregulated in *K. marxianus*, while they were kept unchanged in *S. cerevisiae*. Meanwhile, six out of 12 genes in glycolysis and 14 out of 24 genes in TCA cycle were simultaneously regulated in *K. marxianus* and *S. cerevisiae*, reflecting a general consistency of regulation. In the energy metabolism, 15 out of 16 genes in iron transport and all five genes in heme biosynthesis were upregulated in *K. marxianus*, while they were kept unchanged in *S. cerevisiae*. The results suggested that iron transport and heme biosynthesis were specifically co-upregulated in *K. marxianus*. Out of 47 genes directly involved in the respiratory chain, excluding the genes in ATP synthesis, 14 genes were simultaneously upregulated in *K. marxianus* and *S. cerevisiae*, eight genes were specifically upregulated in *S. cerevisiae* and 18 genes were specifically upregulated in *K. marxianus*. Meanwhile, 14 out of 18 genes involved in the F_O_F1 ATPase-mediated ATP synthesis were specifically upregulated in *K. marxianus*. The results suggested that glucose transport, respiratory chain and ATP biogenesis were particularly co-upregulated in *K. marxianus* during the fast-growing phase.

In a comparison of transcriptomic data of FIM1 cells grown in the medium containing 0 and 4% ethanol (Mo et al., 2019), 14 out of 24 genes in TCA cycles and 13 out of 47 genes in the respiratory chain were upregulated in 4% ethanol, suggesting a weak upregulation of TCA cycle and respiration during ethanol tolerance. In 4% ethanol, 9 out of 17 genes in iron transport and three out of five genes in heme biosynthesis were upregulated, suggesting a weak co-upregulation of both processes (Supplementary Table 15). However, only one out of nine genes involved in glucose transport and 7 out of 17 genes involved in the F_O_F1 ATPase-mediated ATP synthesis were upregulated in 4% ethanol, suggesting a lacking of a general upregulation of both processes (Supplementary Table 15). Therefore, co-upregulation of glucose transport and ATP biogenesis might be a specific feature during the exponential phase in *K. marxianus*.

**FIGURE 7:**
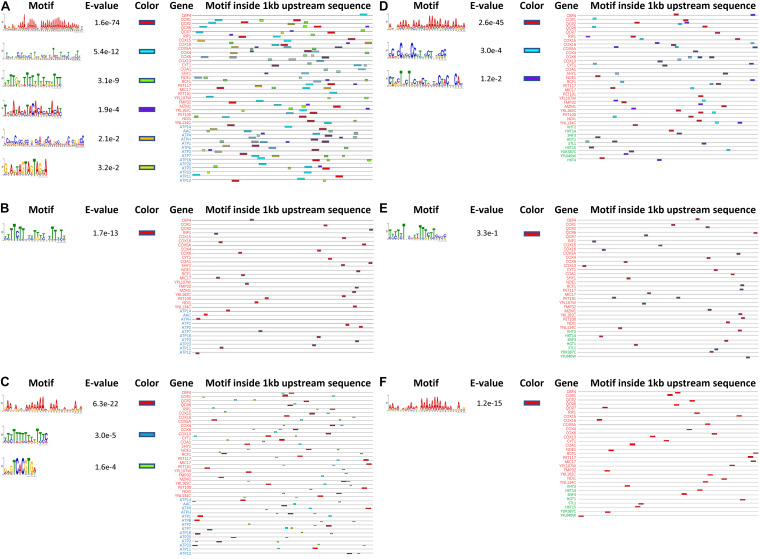
Predicted motifs inside upstream sequences of genes involved in the respiratory chain, ATP synthesis and glucose transport in *K. marxianus*, *S. cerevisiae*, and *K. lactis*. Motifs inside 1 kb upstream sequences of 27 respiratory chain genes (in red) and 14 ATP biosynthesis genes (in blue) are detected in *K. marxianus* **(A)**, *S. cerevisiae* **(B),** and *K. lactis* **(C)**. Motifs inside 1 kb upstream sequences of 27 respiratory chain genes (in red) and 9 glucose transport genes (in green) were identified in *K. marxianus* **(D)**, *S. cerevisiae* **(E),** and *K. lactis* **(F)**. Genes without any identified motif were not shown.

To investigate the underlying mechanism for the simultaneous upregulation of genes in *K. marxianus* during the fast-growing phase, 1 kb upstream sequences of 27 genes in the respiratory chain and those of 14 genes in ATP synthesis from *K. marxianus* and *S. cerevisiae* were analyzed by MEME software online for motif discovery (Bailey et al., 2009). Six significant motifs (*E*-value < 0.05) were located in upstream regions in *K. marxianus* (Figure 7A), and one significant motif was found in *S. cerevisiae* (Figure 7B). Similarly, 1 kb upstream sequence of respiratory chain genes and those of nine genes in glucose transport were analyzed. Three significant motifs were detected in *K. marxianus* (Figure 7D), and only one motif was identified in *S. cerevisiae* (Figure 7E). *K. lactis* is the closest relative of *K. marxianus* in evolution and it also exhibits a fast growth rate comparing to *S. cerevisiae* (Lane and Morrissey, 2010). In *K. lactis*, three significant motifs were detected in upstream sequences of the respiratory chain and ATP synthesis genes (Figure 7C), and one motif was detected in upstream sequences of respiratory chain and glucose transport genes (Figure 7F). Comparing to *S. cerevisiae*, *K. marxianus*, and *K. lactis* harbor increased amount of enriched motifs in upstream sequences of respiratory chain and ATP synthesis genes, suggesting the existence of sequence for the co-regulation of both processes. However, except an A-rich motif, no other consensus motif was found in *K. marxianus* and *K. lactis*. The A-rich motif seemed to be a general feature of upstream sequences in *K. marxianus* and *K. lactis*, as it could also be detected in upstream sequences of glycolysis and TCA cycle genes (Supplementary Figure 2). Therefore, the exact motif or sequence responsible for the co-regulation of respiratory chain and ATP synthesis genes, if it does exist, needs further investigation.

## Discussion

In this study, the comparative genomic and transcriptomic analysis revealed specific features of gene regulation in *K. marxianus*, comparing with *S. cerevisiae*. For example, genes participating in TCA cycles, respiration chain and ATP biosynthesis were specifically upregulated in *K. marxianus* during lag phase (Table 2). Meanwhile, genes involved in glucose transport, including 2 KMS genes (FIM1_7, FIM1_1043) were specifically upregulated in *K. marxianus* during the fast-growing phase (Figures 2, 6). These features might provide some clues to explain the mechanism underlying the well-known fast-growing phenotype of this species.

**FIGURE 8:**
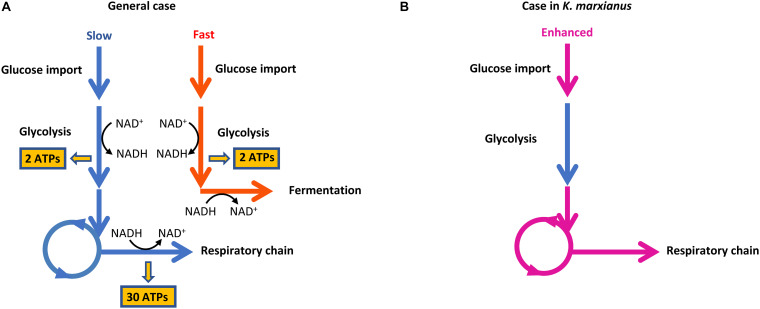
Routes of ATP production in cells. **(A)** The trade-off between ATP production yield and rate. **(B)** Coupling of enhanced glucose transport and respiration chain in *K. marxianus*.

Generally, a higher growth rate relies on higher yield and production rate of ATP (Chen and Nielsen, 2019). However, heterotrophic organisms usually have to face the trade-off between yield and rate of ATP production (Pfeiffer et al., 2001). When substrates with higher free energy are degraded into products with lower free energy, the free energy difference between substrates and products can be partially converted into ATP and be partially used to drive the degradation reaction. For example, if one molecule of glucose is catabolized through the “glycolysis-respiratory chain” route (Figure 8A, blue arrow), 32 molecules of ATPs are generated. In this case, the free energy difference between substrates and products is almost totally preserved in ATPs. The reaction is in thermodynamic equilibrium and low rate of ATP production results in a slow rate of substrate degradation and other reactions such as glucose transport, which hinders the competition with other species for shared resources. If cells adopt the route of “glycolysis-fermentation” for substrate degradation (Figure 8A, red arrow), two molecules of ATPs are produced. The other part of free energy difference can be used to promote the degradation reaction and thus increase the rate of ATP production and glucose transport. This route gains an advantage in the resource competition but low yield ATP can hardly supply sufficient energy for cell growth.

Since *K. marxianus* was considered as the fastest-growing eukaryote on earth (Groeneveld et al., 2009), it must have evolved efficient strategies to reconcile the trade-off between yield and rate of ATP production. We proposed two strategies here that KM complies to improve the efficiency of ATP production, which is required to guarantee its fast growth.

(1) *Kluyveromyces marxianus* simultaneously upregulated respiratory chain and glucose transport, which did not exist in *S. cerevisiae* (Figures 6, 8B). Two KMS genes (FIM1_7, FIM1_1043) proposed to play a role in glucose transport were upregulated during late exponential phase (12 h) (Figure 2). Regarding homologous genes, genes involved in the respiratory chain and glucose transport were co-upregulated in *K. marxianus*, while they were kept unchanged in *S. cerevisiae* during the fast-growing phase (Figure 6). The co-upregulation in *K. marxianus* might be supported by some shared motifs upstream of genes involved in the respiratory chain and glucose transport (Figure 7D). Therefore, upon the reduced rate of glucose transport caused by the low rate of ATP production through respiration pathways, *K. marxianus* might intentionally upregulate the expression of glucose transport genes to promote the absorbance of glucose from the environment. This strategy might alleviate the disadvantage of *K. marxianus* cells in the competition for shared resources during respiration.

(2) *Kluyveromyces marxianus* tightly co-upregulated genes involved in respiratory chain and F_O_F1 ATPase-mediated ATP biosynthesis during the fast-growing phase (Figure 6). In contrast, during the fast-growing phase in *S. cerevisiae*, genes involved in the respiratory chain were upregulated but genes encoding F_O_F1 ATPase were unchanged (Figure 6). The result suggested a relax coupling of the respiratory chain and ATP synthesis in *S. cerevisiae*. The number of shared motifs upstream of the respiratory chain and F_O_F1 ATPase genes in *K. marxianus* (Figure 7A) was greater than that in *S. cerevisiae* (Figure 7B), suggesting that *K. marxianus* optimized the transcriptional regulatory elements to ensure the co-upregulation and coupling of the respiratory chain and ATP synthesis. The coupling improved the efficiency of transformation from free energy difference derived from electron transport chain into ATP and reduced the amount of residual free energy difference which is commonly transformed into heat (Nakamura and Matsuoka, 1978). Heat leads to the stress response and the overexpression of molecular chaperones for protein folding (Gage and Neidhardt, 1993). Consistent with this idea, genes involved in the response to heat and protein folding were upregulated in *S. cerevisiae*, while they were kept unchanged in *K. marxianus* (Table 3).

It should be noted that specific features of gene regulation in *K. marxianus* proposed here was based on the transcriptome of cells cultured in the rich YPD medium under aerobic condition. Rich media supported the fast growth of *K. marxianus* (Rodrussamee et al., 2011), but were not generally applied in industrial fermentation, given the concern of cost reduction. In contrast, fermentation of *K. marxianus* in some poor media was regarded as promising industrial applications. For example, *K. marxianus* was utilized in bioethanol production from cheese whey permeate (Silveira et al., 2005; Zafar and Owais, 2006), and lignocellulosic biomass (Sivarathnakumar et al., 2019). *K. marxianus* was developed to remove copper(II) ions from wastewater in the presence of molasses (Skountzou et al., 2003). The specific growth rates of *K. marxianus* under these conditions reduced dramatically compared with those in rich media. In the future, it will be worthwhile to perform the transcriptomic analysis of *K. marxianus* cells grown in poor media to reveal their specific patterns of gene regulation.

## Conclusion

Comparative genomic analysis revealed 60 KMS genes, grouped in 53 families. 48% of KMS genes were upregulated during cultivation in rich medium and may participate in glucose transport and mitochondrion related functions. Comparative transcriptomic analysis revealed that, during the fast-growing phase of *K. marxianus*, genes participating in respiration chain were specifically co-upregulated with those involved in glucose transport and ATP biosynthesis, which might be attributed to the shared motifs upstream of relevant genes. Specific features in the gene regulation might underly the fast-growing trait of *K. marxianus*. Our study underscores the importance of genome-wide rewiring of the transcriptional network during evolution and proposes a practicable means to explain complex phenotype by combining genome evolution and homologous gene expression analysis.

## Data Availability Statement

The datasets presented in this study can be found in online repositories. The names of the repository/repositories and accession number(s) can be found below: https://www.ncbi.nlm.nih.gov/assembly/GCA_001854445.2; https://www.ncbi.nlm.nih.gov/bioproject/prjna658204.

## Author Contributions

WM, YY, JZh, and JQ performed the genome sequencing and assembly. WM, TL, and JQ carried out the genome annotation and upload to NCBI. XY, HR, and TL carried out the strain cultivation and RNA-seq sample preparation. WL and JQ reconstructed the phylogenetic tree. WM and JQ analyzed the gain/lost genes and high-copied genes. WM, JQ, HL, JZe, and YY analyzed the RNA-seq data. HR performed the RT-qPCR analysis. WM analyzed the motifs of the upstream sequences. YY, WM, and HL prepared the manuscript. HL, JQ, and YY organized this research project. All the authors have read and approved the final version of the manuscript.

## Conflict of Interest

The authors declare that the research was conducted in the absence of any commercial or financial relationships that could be construed as a potential conflict of interest.

## Footnotes

1 https://github.com/tseemann/barrnap

## Supplementary Material

Supplementary Figure 1 Growth curves of *K. marxianus* and *S. cerevisiae*.

Supplementary Figure 2 Predicted motifs inside upstream sequences of glycolysis and TCA cycle genes in *K. marxianus* and *K. lactis*.

Supplementary Table 1 Primers used in this study.

Supplementary Table 2 Correlation between the systematic gene name in FIM1 and DMKU 3-1042 strain.

Supplementary Table 3 Coding sequences of genes in FIM1.

Supplementary Table 4 Gene families in *K. marxianus* and *S. cerevisiae.*

Supplementary Table 5 *K. marxianus*-specific (Kms) genes and families.

Supplementary Table 6 Highly copied genes in *K. marxianus.*

Supplementary Table 7 GFOLD values between expression levels of KMS families at later time points and levels at 1 h.

Supplementary Table 8 Clusters of families which were grouped according to their expression patterns during cultivation.

Supplementary Table 9 Significantly enriched GO terms in the cluster of families.

Supplementary Table 10 GFOLD between expression levels of *K. marxianus* families and those of *S. cerevisiae* families after being cultivated for 1 h.

Supplementary Table 11 Significantly enriched GO terms in relatively upregulated or downregulated families in *K. marxianus* during the lag phase.

Supplementary Table 12 Families ordered by the changes in their expression levels during the fast-growing phase.

Supplementary Table 13 Enriched GO terms in upregulated and downregulated families of *K. marxianu*s or *S. cerevisiae* during the fast-growing phase.

Supplementary Table 14 Gene families grouped with changes of expression levels during the fast-growing phase.

Supplementary Table 15 Changes of mRNA levels during cultivation with ethanol in *K. marxianus*.

Supplementary File 1 GFF file of DMB1 genome.

Supplementary File 2 GFF file of KCTC 17555 genome.

Supplementary File 3 GFF file of CCT 7735 genome.
