# Supplementary material for: Comparative Genomic and Transcriptomic Analysis Reveals Specific Features of Gene Regulation in Kluyveromyces marxianus
Source: Front Microbiol. 2021 Feb 26;12:598060. doi: 10.3389/fmicb.2021.598060 (PMC7953160; doi:10.3389/fmicb.2021.598060)
Supplement: Supplementary Figure 1 — Growth curves of K. marxianus and S. cerevisiae. [file Data_Sheet_1.DOCX]

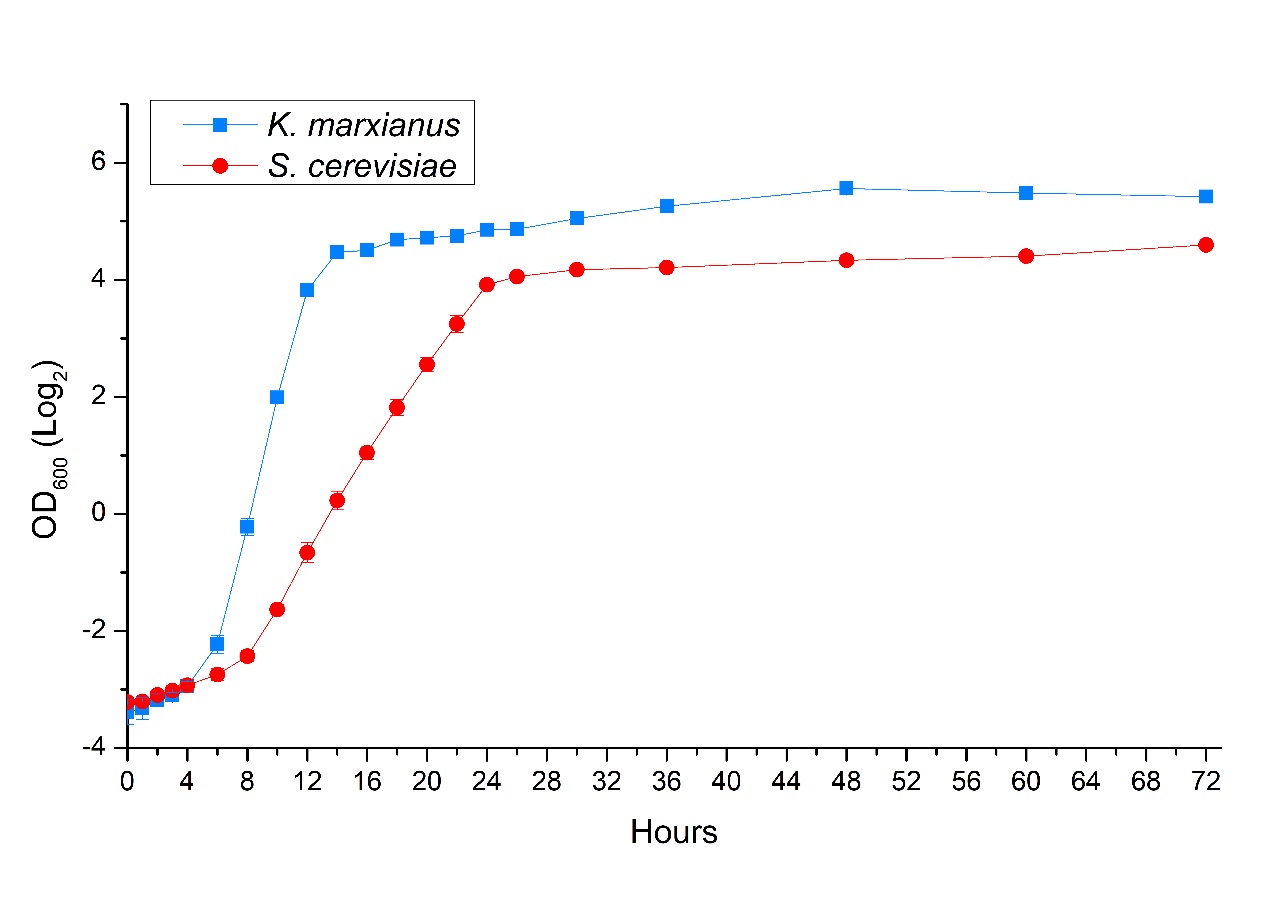


Figure S1. Growth curves of *K. marxianus* and *S. cerevisiae*. Cells were grown in 3ml YPD liquid medium overnight. The culture was transferred to a fresh 50 mL YPD medium in a 150-mL flask to start at an OD_600_ of 0.1. OD_600_ was measured at indicated time points after the start of the culture. The logarithm of OD_600_ to the base 2 was calculated. The value was shown as mean±SD (n=4). The blue curve is for *K. marxianus* and the red curve is for *S. cerevisiae*.
