# Supplementary material for: Comparative Genomic and Transcriptomic Analysis Reveals Specific Features of Gene Regulation in Kluyveromyces marxianus
Source: Front Microbiol. 2021 Feb 26;12:598060. doi: 10.3389/fmicb.2021.598060 (PMC7953160; doi:10.3389/fmicb.2021.598060)
Supplement: Supplementary Figure 2 — Predicted motifs inside upstream sequences of glycolysis and TCA cycle genes in K. marxianus and K. lactis. [file Data_Sheet_2.DOCX]

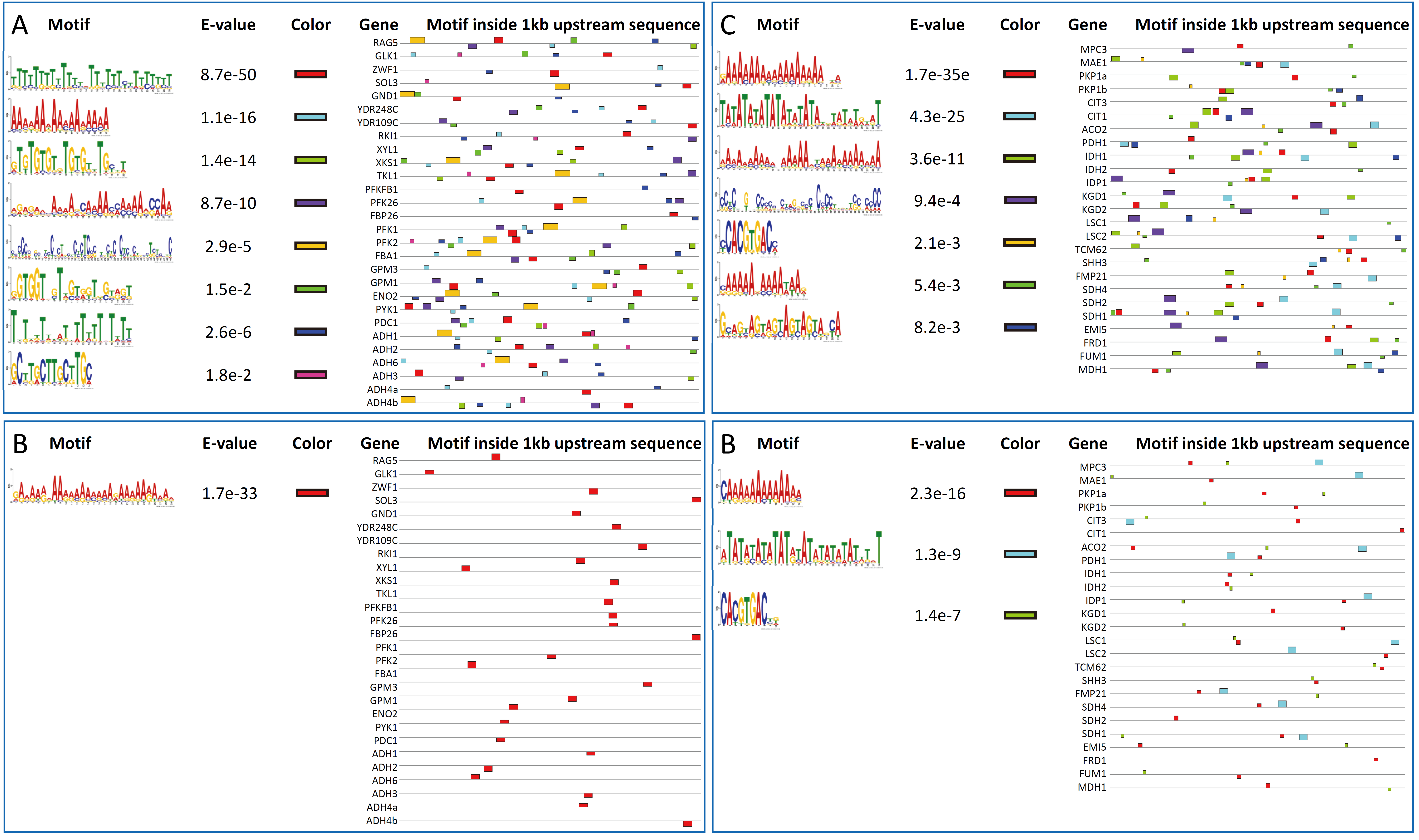


**Figure S2** Predicted motifs inside upstream sequences of glycolysis and TCA cycle genes in *K. marxianus* and *K. lactis*. Motifs inside 1kb upstream sequence of 28 glycolysis genes in *K. marxianus* **(A)** and *K. lactis* **(B)** were predicted by MEME. Motifs inside 1kb upstream sequence of 25 TCA cycle genes in *K. marxianus* **(C)** and *K. lactis* **(D)** were predicted by MEME. Significant motifs (E-value < 0.05) were shown.
